# The Complete Chloroplast Genome of *Idesia polycarpa* and Comparative Analysis of Related Species

**DOI:** 10.3390/genes16050611

**Published:** 2025-05-21

**Authors:** Xueqian Fu, Jie Luo, Yuan Guo, Dalan Feng, Yifei Deng, Mi Kuang, Houlin Zhou, Xia Liu, Chong Sun

**Affiliations:** 1 Chongqing Key Laboratory of Economic Plant Biotechnology, Collaborative Innovation Center of Special Plant Industry in Chongqing, College of Smart Agriculture, Chongqing University of Arts and Sciences, Chongqing 402160, China; fxquick120@163.com (X.F.); luojie@cqwu.edu.cn (J.L.); 15923370686@163.com (Y.G.); 13594098664@163.com (Y.D.); zbgqsc1987@163.com (C.S.); 2 Chongqing Key Laboratory of Forest Ecological Restoration and Utilization in the Three Gorges Reservoir Area, Chongqing Academy of Forestry Sciences, Chongqing 400036, China; fengdalang@163.com; 3 Chongqing Agricultural Technology Promotion General Station, Chongqing 401120, China; kim437@126.com; 4 Chongqing Wulipo National Nature Reserve Management Office, Chongqing 401147, China; z18996669717@163.com; 5 Hubei Key Laboratory of Spices & Horticultural Plant Germplasm Innovation and Utilization, Yangtze University, Jingzhou 434023, China

**Keywords:** *Idesia polycarpa*, genome structure, genetic diversity, phylogenetic analysis

## Abstract

Background/Objectives: The oil grape (*Idesia polycarpa*), often called the “golden tree”, is an essential woody plant valued for its edible oil. Although its economic significance is recognized, the specifics of its chloroplast genome and evolutionary connections remain unclear. This study sequenced the chloroplast genome of *I. polycarpa* and performed a comparative analysis of its genome structure, genetic diversity, and phylogenetics using chloroplast data from related species. Methods: In this study, we sequenced and annotated the whole chloroplast genome of *I. polycarpa* via GISEQ-500 sequencing and de novo assembly. Results: The chloroplast genome of *I. polycarpa* exhibits a typical tetrad structure, with a length of 155,899 bp and a GC content of 36.78%. It comprises 130 unique genes, including 85 coding genes, 37 tRNAs, and eight rRNAs, showing notable conservation in gene composition and arrangement compared to closely related species. However, the inverted repeat region boundaries are narrower. Phylogenetic analysis showed strong relationships among *I. polycarpa*, *Bennettiodendron brevipes*, *Poliothyrsis sinensis*, *Itoa orientalis*, and *Carrierea calycina* within the *Salicaceae* family. Additionally, positive selection analysis revealed that *rpl16*, *ycf1*, *rps18*, and *rpl22* are under significant selective pressure in related species, likely linked to adaptations for photosynthesis and environmental responses. Conclusions: This research provides vital molecular foundations for the conservation, classification, and enhancement of *I. polycarpa* germplasm resources, advancing the study of adaptive evolutionary mechanisms and broadening the genomic database for *I. polycarpa*.

## 1. Introduction

Oil grape, *Idesia polycarpa* Maxim., the only species in the genus *Idesia*, is a high-quality, high-yield woody edible oil tree and an industrial oil raw material tree in China. The species is distributed in northeastern Asia (China, Japan, Korea, Russia). The natural range in China is extensive: the provinces south of the Qinling Mountains and Huai River, including Anhui, Zhejiang, and Jiangxi Provinces. This tree species is highly regarded for its aesthetically pleasing crown structure and robust resistance to various environmental stressors. Furthermore, it is distinguished by its high fruit oil content, earning it the colloquial designation of the ‘tree oil depot’. The content of linoleic acid in the seed of *I. polycarpa* has been reported to be as high as 75%, making it highly valuable for health and nutritional applications [1]. Additionally, this oil serves as a viable raw material for the extraction of linoleic acid preparations and is used in the manufacturing of paints, coatings, and soaps. Given its multifunctional nature, *I. polycarpa* has significant market demand. It is an unusual economic tree species in that it offers value as an ornamental species as well as a source of timber and energy and is thus the focus of much research.

In recent years, with the growing global demand for sustainable resources, the exploration of germplasm resources and the breeding of superior varieties of *I. polycarpa* have become research hotspots. Liu et al. [2] used seeds and leaves of *I. polycarpa* as explants to study the effects of explant types, phytohormones, and other factors on various stages of tissue culture. Their results showed that seeds were the optimal explants for inducing callus formation, and they established a high-frequency tissue culture regeneration system for *I. polycarpa*. Yang et al. [3] investigated the role of explant types and plant growth regulators on organogenesis and plant regeneration under in vitro conditions, identifying the optimal media for subculture and proliferation. Jiang et al. [4] analyzed genetic variation among and within natural populations of *I. polycarpa*, laying a basis for the growth and application of its seed resources and superior variety breeding in China. Jia et al. [5] studied the germination of *I. polycarpa* var. *vestita* Diels seeds at six maturity stages, along with oil content and fatty acid composition in the fruits. Their results revealed significant differences in seed germination rates across maturity stages, providing a scientific basis for determining the optimal harvesting period in production. Fan et al. [6] analyzed the *FAD2* gene to elucidate the molecular mechanisms of fruit oil synthesis in *I. polycarpa*, offering theoretical support for genetic engineering in breeding. Su et al. [7] conducted a diversity analysis of fruit cluster and fruit traits across 16 *I. polycarpa* samples, finding significant differences among samples, which aids in superior variety selection. Cai et al. [8] analyzed the genetic diversity of 373 *I. polycarpa* germplasm resources, exploring correlations among traits and classifying morphological types. Ji et al. [9] investigated dormancy types and optimal methods for breaking dormancy in *I. polycarpa* var. *vestita* seeds, providing efficient germination strategies for seedling production. Wang et al. [10] studied floral characteristics to understand the effect of different flower types and sizes on the growth of trees. Zhang et al. [11] explored the microecological features and internal mechanisms of *I. polycarpa* growth through leaf–soil–microbe interactions, offering scientific insights for improving cultivation practices. Ping et al. [12] analyzed the impact of several drying processes on volatile flavor compounds present in *I. polycarpa* fruits and their oil. Luo et al. [13] focused on assessing the quality features of fruit oil samples from *I. polycarpa* with different phenotypic characteristics, revealing significant differences in oil content among three phenotypic types.

With the widespread adoption of sequencing technologies and advancements in bioinformatics, chloroplast genome data have become a core tool in botanical research due to its unique genetic characteristics (such as maternal inheritance, conserved structure, stable gene arrangement) and functional significance [14]. Pan-organellar genomes have provided new evolutionary insights [15]. Zhou et al. [16] newly assembled 513 complete plastomes of Fagopyrum and aligned with the published plastome of eight *Fagopyrum* species to construct a pan-plastome for *F. tartaricum* and resolve genomic variation. Jia et al. [17] newly assembled the complete chloroplast genomes of 65 *Hemerocallis citrina* Baroni, and conducted a comparative analysis with the published chloroplast genomes of *H. citrina*, the detailed study of the pan-plastome and the examination of the population structure of *H. citrina* plastomes offer crucial information for upcoming breeding initiatives and the conservation of germplasm. Wang et al. [18] assembled 322 complete *Prunus mume* Siebold and Zucc. plastomes and executed a series of comparative analyses to elucidate the pan-plastomic patterns and population structure of *P. mume*; the research results provided a better understanding of the diversity at the genus level and its history, thereby clarifying the complex introgressive relationship between mei and other apricots/plums trees.

Although significant progress has been made in *I. polycarpa* research, its taxonomic classification has long been controversial. Traditional taxonomy places *I. polycarpa* within the Flacourtiaceae family, while phylogenetic trees based on nuclear genome data support its closer relationship to the Salicaceae family (e.g., poplars) [19]. The chloroplast (cp) genome, due to its maternally inherited characteristics, could provide independent evidence for resolving its taxonomic status. For instance, comparative analyses of inversions and repeat events in cp genomes may reveal the divergence pathways between *I. polycarpa* and other Salicaceae species, thereby resolving the long-standing taxonomic disputes. Yang et al. [20] first assembled and characterized the complete cp genome sequence of *I. polycarpa* (Qingchuan, Sichuan Province, China) based on the Illumina pair-end sequencing, thereby enriching the genetic information resource library of this species.

However, research on *I. polycarpa* has mainly focused on its biological [21] and ecological characteristics [22], plant tissue culture [23,24], and fruit and seed chemical composition [5]. There is a paucity of information on its molecular biology, and comprehensive studies of its cp genome are limited. This has seriously hindered the molecular breeding of *I. polycarpa* and the development and utilization of *I. polycarpa* resources. In this study, we sequenced and annotated the complete cp genome of *I*. *polycarpa* and analyzed the structural characteristics. Based on cp genome data, we constructed a phylogenetic tree to clarify its taxonomic position. By comparing the cp genome of *I*. *polycarpa* with those of closely related species, we identified key functional genes potentially under positive selection and elucidated the genetic basis of its environmental adaptation. Furthermore, we can screen highly variable regions or specific genomic fragments to provide a theoretical foundation for subsequent analyses of population genetic diversity, species identification, and resource conservation.

## 2. Materials and Methods

### 2.1. Materials

The fresh leaves of a single individual of *I. polycarpa* were obtained from Hani-Yi Autonomous Prefecture of Honghe (Yunnan, China; 103°34′42.02″ E, 23°6′19.99″ N). This tree grows on the sunny slope of a mountain at an altitude of 1342.3 m. Each sample was rapidly frozen using liquid nitrogen and kept in a lab freezer at −80 °C.

### 2.2. Chloroplast Genome Sequencing, Assembly, and Annotation

The extraction of high-quality total genomic DNA was performed using a Plant Genomic DNA Extraction Kit (Beijing Solarbio Science and Technology Co., Ltd., Beijing, China). After the extracted DNA was detected by agarose gel electrophoresis, a library suitable for the sequencing platform was constructed. The constructed library was sequenced using a GISEQ-500 sequencer (Huada Manufacturing Technology Co., Ltd., Shenzhen, China), and the PE150 reads were long. After sequencing, clean reads were used for de novo assembly of cp genomes using SPAdes Assembler (v3.9.0) [25].

GeSeq [26] and ARAGORN [27] were used for genome-wide annotation. To increase the accuracy of the annotation results, Geneious (v9.1.8) [28] was used for manual proofreading to check the differential genes between the two annotation results, remove errors and redundancies, and obtain the final annotation. The newly annotated cp genome data of *I. polycarpa* was sent to GenBank (PV495210).

### 2.3. Chloroplast Genome Characteristics

The GC content of the cp genome sequence was determined using EditSeq (v7.1.0) software [29]. The OGDRAW (v1.3.1) software [30] was used to draw the cp genome circle of *I. polycarpa*.

### 2.4. Repeat Sequence and Simple Repeat Sequence Analysis

Using REPuter [31] online tool (https://bibiserv.cebitec.uni-bielefeld.de/reputer, accessed on 3 May 2025), the cp genomes of *I. polycarpa* and six closely related species (*Populus davidiana*, *Salix linearistipularis*, *Bennettiodendron brevipes*, *Poliothyrsis sinensis*, *Itoa orientalis*, *Carrierea calycina*) were documented. Five repeating sequences were found, including forward, reverse, palindromic complement, and tandem sequences. The maximum number of repeats was set to 100 bp, the minimum repeat size was 30 bp, and the Hamming distance was 3. SSR locus information of the cp genome sequences of the seven species was analyzed using the MISA online tool [32] (https://webblast.ipk-gatersleben.de/misa/index.php, accessed on 3 May 2025). For mononucleotide, dinucleotide, trinucleotide, tetranucleotide, pentanucleotide, and hexanucleotide, the values were set at 10, 5, 4, 3, 3, and 3, respectively, and the minimum gap between two SSRs was established at 100 bp.

### 2.5. Analysis of Codon Preference

The software codonW (v1.4.4) [33] was used to detect the number of codons and the relative synonymous codon usage (RSCU) of synonymous codons in *I. polycarpa* and six closely related species, and the RSCU values were visualized.

### 2.6. Analysis of IR Boundary

IRscope (v3.1) [34] software was used to detect the shrinkage and expansion of the LSC, SSC, and IRs region boundaries in the genomes of *I. polycarpa* and the six closely related species.

### 2.7. Analysis of Selection Pressure 

Ka and Ks calculations were performed using the *I. polycarpa* sequence as the reference sequence and six closely related species sequences, respectively. First, the coding and protein sequences of the cp genome of *I. polycarpa* were extracted using the PhyloSuite (v1.2.3) software. BLASTN v2.10.1 software was used to compare the protein sequences of six related species with the protein sequences of *I. polycarpa* to find the best match to obtain homologous protein sequences. Automatic alignment of homologous protein sequences using MAFFT (v7.526) software [35]. The perl script was used to map the aligned protein sequence to the coding sequence to obtain the aligned coding sequence, and the KaKs_Calculator (v3.0) [36] is used to calculate the Ka and Ks values.

### 2.8. Comparative Analysis of CP Genomes

The mVISTA online tool [37] (http://genome.lbl.gov/vista/mvista/submit.shtml, accessed on 3 May 2025) was used to select seven genomes for visual analysis in Shuffle-LAGAN mode. DnaSP (v.6) [38] was employed to determine the nucleotide diversity (Pi) of the genome sequence, and highly variable regions in the genome were screened out. The parameters were set with a sliding window of 600 and a step size of 200.

### 2.9. Phylogenetic Relationship Analysis

The genome sequences of 27 species were used, including one newly sequenced *I. polycarpa*, six Salicaceae species downloaded from NCBI, 18 Flacourtiaceae species, and two Linaceae species. Phylogenetic trees were built using these cp genome sequences, and the Linaceae family species were used as the outgroup. MAFFT was used to compare the cp genome sequences of 27 species, and MEGA11 was employed to build a phylogenetic tree. The bootstrap value was set to 1000 with LG + G as the best model.

## 3. Results

### 3.1. Chloroplast Genome Assembly and Genome Features

**Figure 1:**
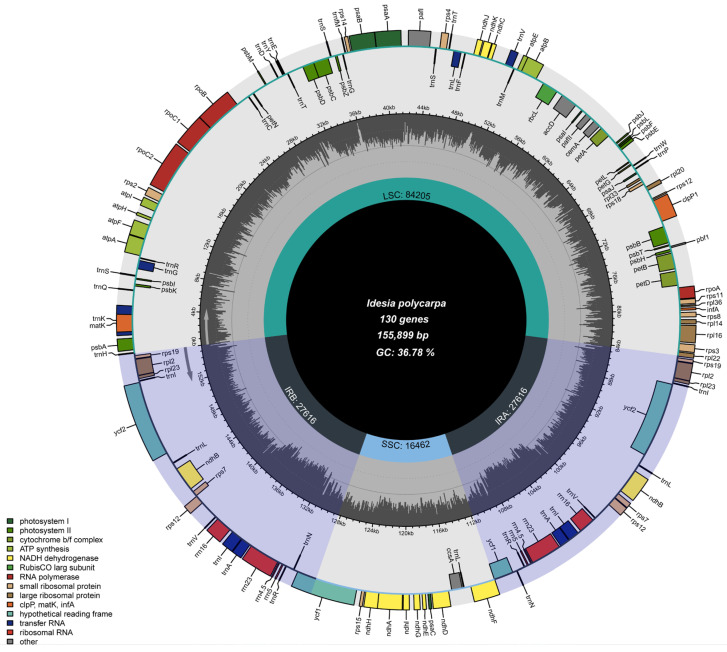
Gene map of the complete cp genome of *Idesia polycarpa*. The cp genome gene in the circle is transcribed clockwise, and the cp genome gene outside the circle is transcribed counter-clockwise. Different colors are used to encode genes from distinct functional groups. The inner ring’s deeper gray signifies the GC content. Shown are small single copy (SSC) regions, large single copy (LSC) sequences, and inverted repeats (IRa and IRb).

The cp genome of *I. polycarpa* has a typical quadruple structure (Figure 1). The total length of the genome sequence is 155,899 bp, including a large single copy region (LSC) of 84,205 bp, a small single copy region (SSC) of 16,462 bp, and two inverted repeat regions (IRa and IRb) with a combined length of 27,616 bp. The GC content of the cp genome was 36.78%, of which the CG content of the two IR regions was the highest, reaching 41.88%, the LSC region was 34.62%, and the SSC region was the lowest, 30.68%.

The annotation included 130 genes in total, with 85 being protein-coding, 37 tRNA, and eight rRNA genes (Table 1). A total of 21 genes contained introns in the whole sequence: *trnK-UUU*, *trnG-UCC*, *atpF*, *rpoC1*, *trnL-UAA*, *trnV-UAC*, *rps12*, *rps12-2*, *petB*, *rpl16*, *rpl2*, *ndhB*, *trnI-GAU*, *trnA-UGC*, *ndhA*, *trnA-UGC-2*, *trnI-GAU-2*, *ndhB-2*, *rpl2-2;* a total of 19 genes contained one intron; *clpP1* and *pafI* contained two introns; and *petD* contained only two exons. Twelve cis-splicing genes were identified, including *atpF*, *rpoC1*, *pafI*, *clpP1*, *petB*, *petD*, *rpI16*, *rpl2* (two-copy regions), *ndhA* and *ndhB* (two-copy regions) (Table 2).

### 3.2. Repeat Sequences and SSR Analysis

**Figure 2:**
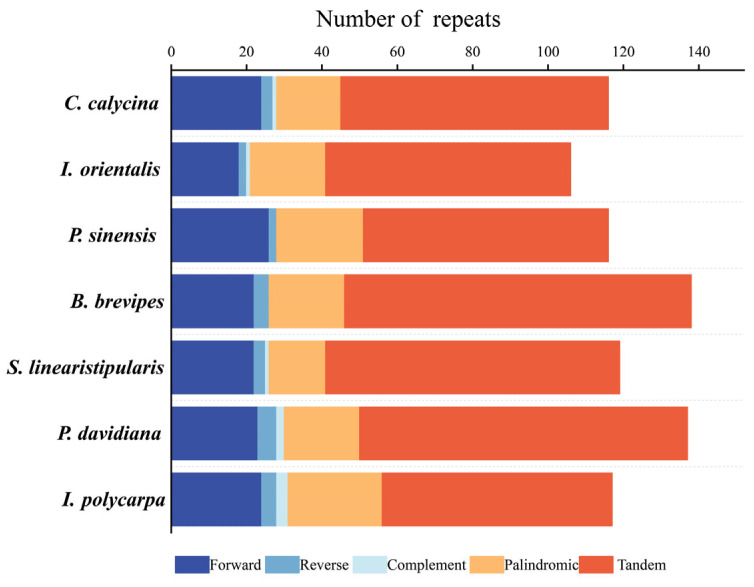
Number and types of long repeats.

A total of 330 long repetitive sequences were identified in the cp genomes of *I. polycarpa*, *P. davidiana*, *S. linearistipularis*, *B. brevipes*, *P. sinensis*, *I. orientalis*, *C. calycina.* The sequences included 159 forward repeats (F), 23 reverse repeats (R), eight complement repeats (C), and 140 palindromic repeats (P). Comparing the long repeat numbers and types in the cp genomes of seven different species. The results showed that the long-repeat types of *I. polycarpa*, *P. davidiana*, and *S. linearistipularis* were the same, and the number of long repeats was similar. The number of long repeats in *I. polycarpa* was 24F, 4R, 3C, 25P; *P. davidiana* was 23F, 5R, 2C, 20P; *S. linearistipularis* was 22F, 3R, 1C, 15P. In addition, the four closely related species, *I. polycarpa*, *B. brevipes*, *P. sinensis*, *I. orientalis*, *C. calycina* are also extremely close in the number of long repeats. *B. brevipes* and *P. sinensis* did not contain any complementary repeats. In addition, 519 tandem repeats (T) were detected in the seven species: *I. polycarpa* 61T, *P. davidiana* 87T, *S. linearistipularis* 78T, *B. brevipes* 92T, *P. sinensis* 65T, *I. orientalis* 65T, *C. calycina* 71T (Figure 2).

**Figure 3:**
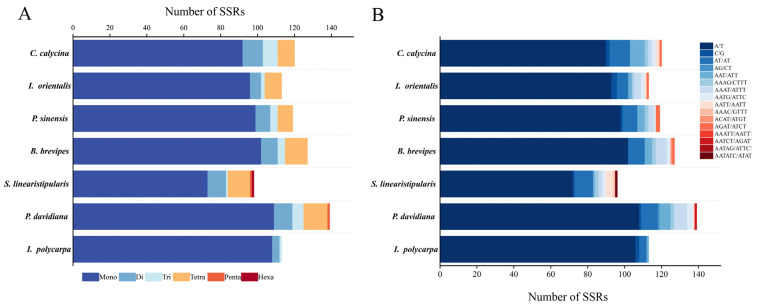
Number and types of nucleotide repeats. (**A**) Simple repeat sequence identification statistics. (**B**) Type and number of simple repetitions.

MISA detected SSRs in the cp genomes of the seven species, and 851 SSRs were detected. The number of *P. davidiana* SSRs was the highest (139), followed by *B. brevipes* (127), *C. calycina* (120), *P. sinensis* (119), *I. orientalis* (113), and *I. polycarpa* (113). The number of SSRs detected by *S. linearistipularis* was the lowest (98). Six types of nucleotide repeats were detected (Figure 3), including 679 mononucleotides, 58 dinucleotides, 26 trinucleotides, 63 tetranucleotides, two pentanucleotide repeats, and one hexanucleotide repeat. The SSRs of each species were mainly single-nucleotide repeats (72–109), accounting for 81.91% of the total SSRs, followed by trinucleotide SSRs, which were less abundant than dinucleotide and tetranucleotide SSRs, whereas pentanucleotide and hexanucleotide sequences were the least abundant; only one hexanucleotide was detected in *S. linearistipularis*.

### 3.3. Codon Usage of Cp Genome

**Figure 4:**
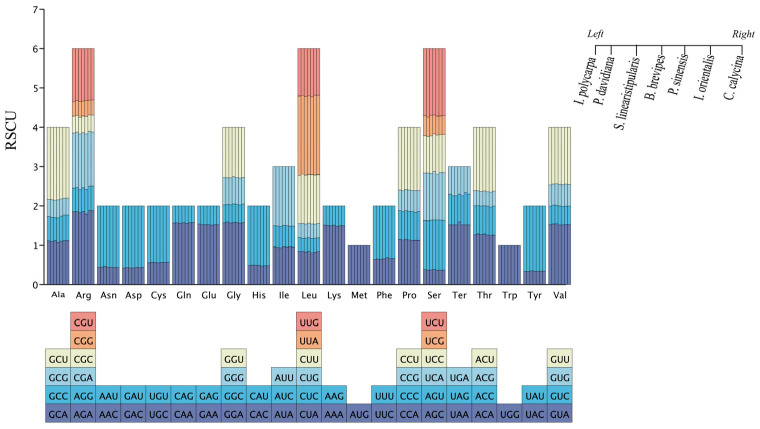
The protein-coding genes of the seven cp genomes were identified based on codon preferences and amino acid ratios of relatively synonymous codon usage (RSCU). Ter denotes the termination codon and the right side represents the ordinate species.

The relative synonymous codon usage values (RSCUs) of seven cp genome-coding regions of *I. polycarpa* and its related species were analyzed, and codon usage frequency and preference in the genome were detected (Figure 4). The sequenced genome encodes 21 amino acids with 22,703, 22,665, and 22,613 codons in *I. polycarpa*, *P. davidiana*, and *S. linearistipularis*, respectively. There were 22,761, 21,599, 22,764, and 22,757 codons in *B. brevipes*, *P. sinensis*, *I. orientalis*, *C. calycina*, respectively (including stop codons). Among the encoded amino acids of all species, leucine (Leu), serine (Ser), and arginine (Arg) accounted for 10.62–10.78%, 7.47–7.55%, and 5.79–5.85% of the total codons, respectively, and methionine (Met) 2.33–2.52% and tryptophan (Trp) 1.74–1.78% were the least.

In addition, the relative synonymous codon usage (RSCU) values ranged from 0.3325 to 2.0297, and most of the codons ended with A/U (A/T) (70.59–71.2%), which had a preference for A/U (A/T) bases. Arginine (Arg), leucine (Leu), and serine (Ser) have high codon preferences in individual species. Only two codons (UGG and AUG) had an RSCU of 1, indicating that methionine (Met) and tryptophan (Trp) had no codon preference.

### 3.4. IR Boundary Analysis

**Figure 5:**
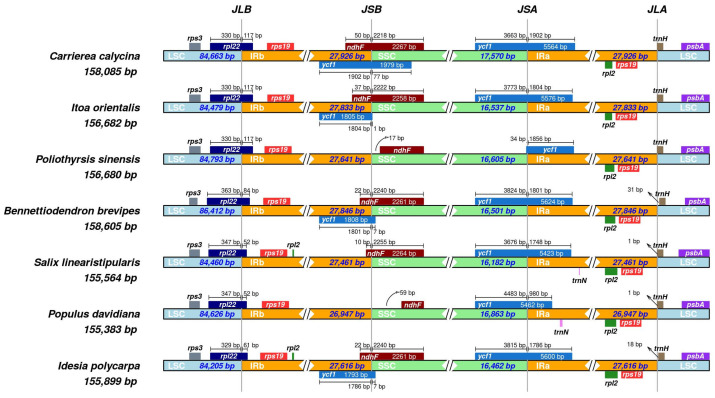
Comparative analysis of the LSC, SSC, and IR boundaries of the cp genomes of seven species.

Many species commonly exhibit contraction/expansion in the boundaries of the IR region. By comparing the IR region boundaries of different species, similarities and differences between species can be identified, which canenrich genetic data for phylogenetic analysis to provide a basis for plant classification and evolutionary research. Therefore, the IR region boundaries of the cp genomes of the seven species were compared and analyzed (Figure 5).

The study showed that the cp genomes of the seven species were conserved; however, there were some differences. The IR regions of *I. polycarpa* was 27,616 bp, *S. linearistipularis* IR region was 27,461 bp, *P. davidiana* IR region was 26,947 bp, respectively. The IR region showed a contraction phenomenon compared with the other four closely related species. Among the seven species, *rps3*, *rpl22* and *rps19* located at the boundary of the LSC/IRb region were the same, among which *rpl22* showed obvious differences. *P. sinensis*, *I. orientalis*, and *C. calycina* had the same size and location as *rpl22*, occupying 330 bp in the LSC, 117 bp in the IRb, and *rpl22* in *B. brevipes*. The size of *rpl22* was the same as that in the other three species. However, there was a significant contraction, occupying only 84 bp in IRb. The size and position of *rpl22* in *P. davidiana* and *S. linearistipularis* were very similar, occupying 329 bp and 347 bp in LSC and 61 bp and 52 bp in IRb, respectively. Compared with the other four species, the contraction was more intense. At the boundary of the IRb/SSC region, all seven species have *ndhF* genes, but they are closer to the SSC region, showing a shrinkage phenomenon. At the two ends of IRa, *ycf1*, *rpl2*, *rps19*, *trnH*, and *psbA* were detected in all seven species. By observing ycf1 and *trnH*, it was found that *trnH* genes were located in the LSC region. *P. davidiana* and *S. linearistipularis* were 1 bp from the IRa boundary, and *I. polycarpa* and *B. brevipes* were 18 and 31 bp from the IRa boundary, respectively. In addition, *ycf1* is closer to the SSC region, which can be further determined, and the IR region shrinks.

### 3.5. Comparative Analysis of Cp Genomes

**Figure 6:**
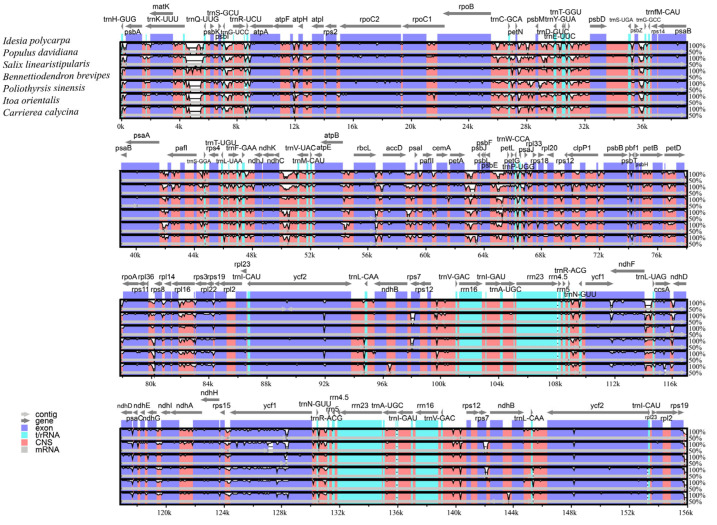
The mVISTA software was used to compare the cp genomes of the seven species. The gray arrow and thick black line above the alignment represent the direction and IR position of the gene, respectively. The Y axis represents the similarity percentage (50–100%). The color coding of the genomic region is protein coding (exon, purple), ribosomal RNA (rRNA, cyan), and conserved noncoding sequences (CNS, pink).

The mVISTA tool was used to determine the degree of difference between the cp genome sequences of the seven species. The other six sequences were compared with the reference sequence of *I. polycarpa* (Figure 6). The results showed a certain degree of similarity among the seven sequences. Among them, the degree of variation of the IR region was lower than that of the other regions, and the degree of variation of the non-coding region was significantly higher than that of the coding region.

**Figure 7:**
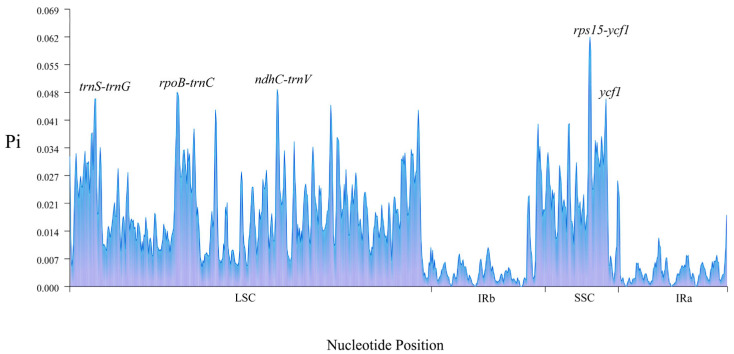
Comparison of nucleotide variability (Pi) values of the seven cp genomes. The Y-axis represents the Pi value and the X-axis represents the region of the gene.

The cp genome nucleotide diversity (Pi) of the seven species was analyzed using DNAsp software. A total of 724 polymorphic sites were detected, with Pi values of 0.00048–0.06175 and an average Pi value of 0.01463. Additionally, five regions with high nucleotide variation rates (Pi > 0.046) were screened out, which were *rps15-ycf1*, *ycf1*, *ndhC-trnV*, *rpoB-trnC*, *and trnS-trnG*, *trnG*. Five hypervariable regions were located in the LSC and SSC, and the Pi value of *rps15-ycf1* in the intergenic region was 0.06175 (Figure 7).

### 3.6. Selection Pressure Analysis

**Figure 8:**
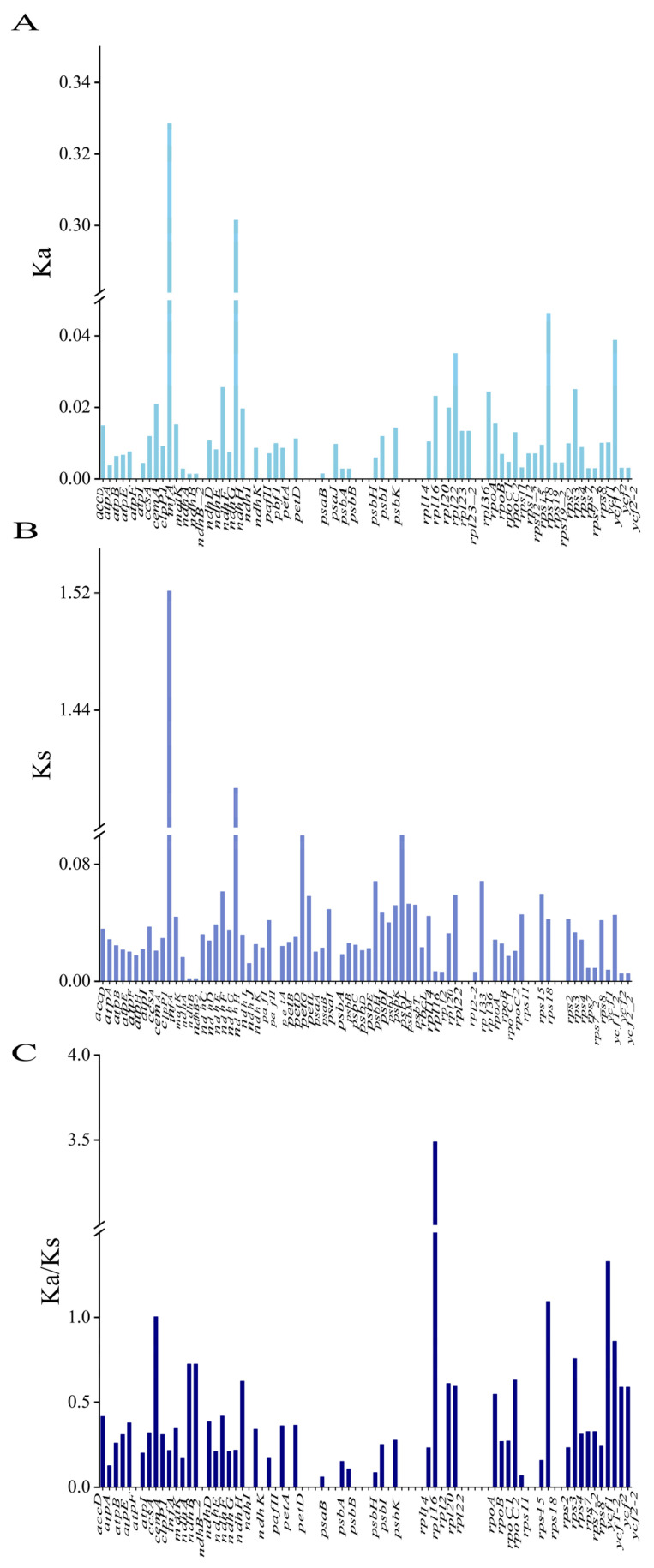
Selection pressure for 80 protein-coding genes in the cp genomes of six related species and *Idesia polycarpa*. (**A**) Ka, synonymous substitution rate; (**B**) Ks, non-synonymous substitution rate; (**C**) Ka/Ks, non-synonymous and synonymous substitution rates.

Synonymous (Ks) and nonsynonymous (Ka) substitution rates were analyzed using 80 protein-coding genes in the cp genomes of the seven species. The Ka/Ks ratios of all protein-coding genes ranged from 0 to 3.48857, with an average of 0.27. The Ka/Ks ratio of *rpl16* was the highest at 3.48857. In addition, the genes with Ka/Ks > 1 were *ycf1*, *rps18*, and *cemA*, with Ka/Ks values of 1.32814, 1.09266, and 1.00319, respectively (Figure 8C).

### 3.7. Phylogenetic Analysis

**Figure 9:**
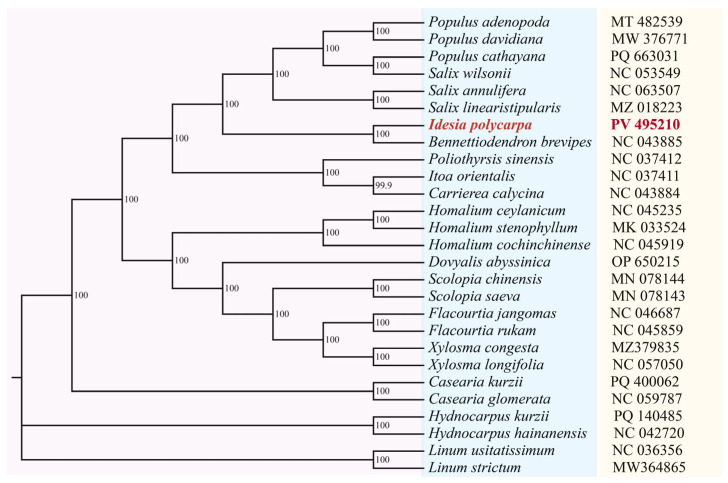
A phylogenetic tree comprising 27 species was constructed. The species names marked in red color is the newly sequenced species identified in this study, the latter is the GeneBank accession number.

The results showed that the maximum likelihood (ML) phylogenetic tree constructed with common protein-coding gene sequences was highly supported (Figure 9). The two species of *Linum usitatissimum* and *Linum strictum* of the Linaceae form independent branches as outgroups. Two species of *Hydnocarpus* genus in the Flacourtiaceae diverged first, followed by two species of *Casearia* in the Flacourtiaceae. Every species within the Salicaceae family formed a single monophyletic group, and sister groups with *I. polycarpa* and *B. brevipes*, followed by *P. sinensis*, *I. orientalis*, and *C. calycina*. The remaining Flacourtiaceae species were clustered into one branch with a relatively high support value (PP = 100).

## 4. Discussion

### 4.1. Structural Features of the Cp Genome in Idesia polycarpa

This study involved the successful sequencing, assembly, and annotation of the entire cp genome sequence of a species of *I. polycarpa* and further analyzed its genomic characteristics, GC content, and gene structure. According to the results, the cp genome of *I. polycarpa* featured a typical tetrad configuration, with a total length of 155,899 bp, including a large single copy region (LSC) of 84,205 bp, a small single copy region (SSC) of 16,462 bp, and two inverted repeat regions (IRa and IRb) with a combined length of 27,616 bp. This result differs from that reported by Yang et al. [20], who documented a total chloroplast genome length of 157,017 bp for *I. polycarpa* samples from Qingchuan, Sichuan, China, with the LSC region measuring 84,787 bp, the SSC region 16,512 bp, and the IRs 27,859 bp each. This discrepancy could be due to the substantial genetic variation among *I. polycarpa* samples from different geographic provenances [4]. The GC content in the IR region was higher than that in the other regions (LSC and SSC), which is a common phenomenon in plants. The higher GC content in the IR region is mainly attributed to the abundance of rRNA and tRNA genes [39]. The annotation included 130 genes in total, with 85 being protein-coding, 37 tRNA, and eight rRNA genes (Table 1). This finding aligns with the total gene number of *I. polycarpa* reported by Yang et al. [20].

It is worth noting that the functions of the three genes, *pafI*, *pafII*, *pbf1* in the sequence have not been fully defined and are rarely mentioned in other studies. However, most of the sequences surrounding these three genes were tRNAs. The arrangement of this gene position may imply that it has specific functions in transcription, RNA processing, or translation regulation. Although the specific functions of these genes are not yet clear, the plant cp genome is highly conserved, indicating that they may play important roles in cp functions. It may also be a unique gene carried by the chloroplasts of *I. polycarpa*. Additional research is required to confirm the roles of these genes and explore their significance in plant physiology and evolution.

### 4.2. Phylogenetic Relationship Between Idesia polycarpa and Related Species

Due to the conserved structure and slow evolution of plant cp genomes, species identification and phylogenetic evolution of cp genomes have become the developmental trend in plant taxonomic biology and have attracted increasing attention and recognition from researchers [40]. Simple sequence repeats (SSRs) play important roles in many applications, including species identification, population genetics, and phylogenetic research [14,41]. Through the study of cp genes, it was found that the number of single-nucleotide repeats was the highest in the SSRs sequence of the cp genome. This phenomenon is also true for the cp genomes of *I. polycarpa* and six related species, which contribute to A/T richness. These results are consistent with those reported for most angiosperms [42,43]. The number of tetranucleotides was slightly higher than the number of dinucleotides. There were 63 tetranucleotides, 58 dinucleotides, and 26 trinucleotides, indicating that dinucleotide and tetranucleotide repeats may provide stronger adaptive advantages or play important roles in genome stability. Long repeat sequence analyses identified 159 forward repeats and 140 palindromic repeats in the seven species. These repeat sequences may have been retained during evolution, indicating that they are important for species adaptability and survival. Codons play important roles in protein synthesis, gene regulation, and evolutionary biology [44,45]. Among the seven species, leucine (Leu), serine (Ser), and arginine (Arg) accounted for 10.62–10.78%, 7.47–7.55%, and 5.79–5.85% of the total codons, respectively. These are common amino acids that play important roles in the structure and function of photosynthesis-related proteins. Methionine (Met) and tryptophan (Trp) did not show codon preferences.

Although IR are more conserved in the cp genome than in the SSC and LSC regions, the evolutionary events and size changes in the cp genomes across various plants can also be linked to the expansion and contraction of IR boundaries [46,47,48]. In this study, the genes *rpl22*, *ndhF*, *ycf1*, and *trnH* at the LSC/IRb-IRa and SSC/IRb-IRa boundaries were clearly concluded to have shrunk in the IR region, whereas at the IRB/SSC boundary, *P. davidiana*, *S. linearistipularis*, *P. sinensis* species did not have *ycf1*, which may have undergone genomic recombination events during evolution, resulting in these genes being removed from the IRB/SSC boundary or relocated to other regions.

The Ka/Ks ratio is related to gene adaptive evolution, which is a meaningful marker in species evolution [49,50]. The Ka/Ks ratio can explain gene selection. In practice, some genes can be screened based on their Ka/Ks ratios for further functional studies. The Ka/Ks ratio has been widely used to study molecular evolution [51]. In the present study, the results of synonymous (Ks) and nonsynonymous (Ka) substitution rate analyses showed that Ks was much higher than Ka, indicating that the evolution of the seven species was relatively slow. The Ka and Ks values of *infA* were relatively high at 0.328445 and 1.52127, respectively. The average Ka/Ks ratio of *rpl16* was the highest, indicating strong positive selection. This gene may be rapidly evolving. However, their functions require further investigation. Similar to findings from previous research, genes involved in photosynthesis evolve more slowly than other protein-coding genes [52,53,54]. We identified five hypervariable regions, *rps15-ycf1*, *ycf1*, *ndhC-trnV*, *rpoB-trnC*, and *trnS-trnG* by comparing the structures of seven cp genomes and analyzing nucleotide polymorphisms (Pi). Specific molecular markers for identifying *I. polycarpa* could be developed using these five highly variable regions.

In the traditional classification, plants of Flacourtiaceae belong to a morphologically diverse group containing approximately 90 genera and 800–1000 species, widely distributed in tropical and subtropical regions. Its morphological characteristics are highly heterogeneous, resulting in disputes over the taxonomic affiliations of many genera within this family. Some Flacourtiaceae species (such as *Idesia* and *Xylosma*) have morphological features (such as unisexual flowers and capsules) similar to those of Salicaceae, leading researchers to speculate on the potential phylogenetic relationships between these two groups [19]. However, there is a lack of clear evidence supporting this speculation. With the development of molecular systematics, the application of DNA sequence analysis techniques (such as *rbcL*, *matK*, *ITS* transcribed spacer) has shown that the Flacourtiaceae family is not a monophyletic group. Its members are scattered across different branches of Malpighiales and some genera have close phylogenetic relationships with Salicaceae. However, previous studies were mainly based on a few short gene fragment sequences, which contained limited phylogenetic information and could not resolve interspecific phylogenetic relationships [55]. In the present study, we constructed a phylogenetic tree based on complete cp genome sequences and clarified the disputed interspecific relationships among several genera of Flacourtiaceae. This further verified that the core genera of Flacourtiaceae, such as *I. polycarpa*, *B. brevipes*, *P. sinensis*, *I. orientalis*, and *C. calycina*, had relatively close phylogenetic relationships with the Salicaceae, and the branch had a relatively high support rate (Figure 9). The phylogenetic analysis showed that *I. polycarpa* is closely related to the genera of *Salix* and *Populus*, consistent with the findings of Yang et al. [20]. These results are consistent with the official split of the Flacourtiaceae family by the APG IV classification system, where its core genera were incorporated into Salicaceae, and other genera were dispersed to different families within Malpighiales (such as Achariaceae and Salicaceae sensu lato).

## 5. Conclusions

The study is the first to reveal the structural characteristics of the cp genome of *I. polycarpa* and related species, along with the phenomenon of IR contraction. Based on the cp genome data, the classification status of *I. polycarpa* has been clarified, providing molecular evidence for the taxonomic disputes regarding the species. In the future, we will explore the molecular mechanisms causing the differences among various varieties of *I. polycarpa*, offering molecular-level support for variety selection and breeding. The five highly variable regions identified in this study lay the foundation for functional genomics and the development of molecular markers for *I. polycarpa*. We will analyze the functions of genes such as *ycf1*, *rps18*, *cemA*, and *rpl16*, and their roles in environmental adaptation, combined with population genetics, to investigate the association between the polymorphism of SSR loci and geographical distribution, thereby contributing to the efficient utilization and industrial upgrading of *I. polycarpa* resources.

## Figures and Tables

**Table 1:** Gene composition in cp genome of *Idesia polycarpa*.

Category	Gene Group	Gene Name	Number
Photosynthesis	Subunits of photosystem I	*psaA*, *psaB*, *psaC*, *psaI*, *psaJ*	5
	Subunits of photosystem II	*psbA*, *psbB*, *psbC*, *psbD*, *psbE*, *psbF*, *psbH*, *psbI*, *psbJ*, *psbK*, *psbL*, *psbM*, *psbT*, *psbZ*	14
	Subunits of NADH dehydrogenase	*ndhA* *, *ndhB* *(2), *ndhC*, *ndhD*, *ndhE*, *ndhF*, *ndhG*, *ndhH*, *ndhI*, *ndhJ*, *ndhK*,	12
	Subunits of cytochrome b/f complex	*petA*, *petB* *, *petD* *, *petG*, *petL*, *petN*	6
	Subunits of ATP synthase	*atpA*, *atpB*, *atpE*, *atpF* *, *atpH*, *atpI*	6
	Large subunit of rubisco	*rbcL*	1
	Subunits photochlorophyllide reductase	——	
Self-replication	Proteins of large ribosomal subunit	*rpl14*, *rpl16* *, *rpl2* *(2), *rpl20*, *rpl22*, *rpl23(2)*, *rpl33*, *rpl36*	10
	Proteins of small ribosomal subunit	*rps11*, *rps12* **(2), *rps14*, *rps15*, *rps18*, *rps19(2)*, *rps2*, *rps3*, *rps4*, *rps7(2)*, *rps8*	14
	Subunits of RNA polymerase	*rpoA*, *rpoB*, *rpoC1* *, *rpoC2*	4
	Ribosomal RNAs	*rrn16(2)*, *rrn23(2)*, *rrn4.5(2)*, *rrn5(2)*	8
	Transfer RNAs	*trnA-UGC* *(2), *trnC-GCA*, *trnD-GUC*, *trnE-UUC*, *trnF-GAA*, *trnG-GCC*, *trnG-UCC* *, *trnH-GUG*, *trnI-CAU(2)*, *trnI-GAU* *(2), *trnK-UUU* *, *trnL-CAA(2)*, *trnL-UAA* *, *trnL-UAG*, *trnM-CAU*, *trnN-GUU(2)*, *trnP-UGG*, *trnQ-UUG*, *trnR-ACG(2)*, *trnR-UCU*, *trnS-GCU*, *trnS-GGA*, *trnS-UGA*, *trnT-GGU*, *trnT-UGU*, *trnV-GAC(2)*, *trnV-UAC* *, *trnW-CCA*, *trnY-GUA*, *trnfM-CAU*	37
Other genes	Maturase	*matK*	1
	Protease	*clpP1* **	1
	Envelope membrane protein	*cemA*	1
	Acetyl-CoA carboxylase	*accD*	1
	c-type cytochrome synthesis gene	*ccsA*	1
	Translation initiation factor	*infA*	1
	other	*pafI* **, *pafII*, *pbf1*	3
Genes of unknown function	Conserved hypothetical chloroplast ORF	*ycf1(2)*, *ycf2(2)*	4

* Gene with one intron; ** gene with two introns; Gene (2): number of copies of multi-copy genes.

**Table 2:** Intron-containing genes and their exons and intron lengths in the cp genome of *Idesia polycarpa*.

Gene	Location	Exon1	Intron1	Exon2	Intron2	Exon3
*trnK(UUU)*	LSC	37	2544	35		
*trnG(UCC)*	LSC	23	708	48		
*atpF*	LSC	145	734	410		
*rpoC1*	LSC	432	776	1617		
*pafI*	LSC	124	722	230	712	153
*trnL(UAA)*	LSC	35	581	50		
*trnV(UAC)*	LSC	39	594	35		
*rps12*	LSC + IRa	114	536	232		26
*rps12-2*	LSC + IRb	114	536	232		26
*clpP1*	LSC	71	762	292	640	228
*petB*	LSC	6	814	642		
*petD*	LSC	8		490		
*rpl16*	LSC	9	1102	399		
*rpl2*	IRa	391	668	434		
*ndhB*	IRa	777	682	756		
*trnI(GAU)*	IRa	37	948	35		
*trnA(UGC)*	IRa	38	809	35		
*ndhA*	SSC	553	1033	545		
*trnA(UGC)-2*	IRb	38	809	35		
*trnI(GAU)-2*	IRb	37	948	35		
*ndhB-2*	IRb	777	682	756		
*rpl2-2*	IRb	391	668	434		

## Data Availability

The data presented in this study are openly available in GeneBank.
